# Rectal pulse granuloma: a rare condition presenting as a subepithelial lesion

**DOI:** 10.1055/a-2351-3633

**Published:** 2024-07-08

**Authors:** Cong Yuan, Xue-Mei Lin, Chun-Hui Xi, Dan Sun, Xiao-Bo Wang, Guo-Dong Yang, Xian-Fei Wang

**Affiliations:** 1 117913 Gastroenterology and Digestive Endoscopy Center, Affiliated Hospital of North Sichuan Medical College, Nanchong, China; 2 74655 Pathology, Institute of Basic Medicine and Forensic Medicine, North Sichuan Medical College, Nanchong, China; 3 117913 Pathology, Affiliated Hospital of North Sichuan Medical College, Nanchong, China

A pulse granuloma is a rare benign entity that typically occurs in the oral cavity 1 . Herein, we report a case of a subepithelial lesion (SEL) located in the distal rectum, which was diagnosed as a rectal pulse granuloma after its removal by endoscopic submucosal dissection (ESD).

A 66-year-old man with no significant medical history underwent colonoscopy for adenoma screening. Colonoscopy revealed multiple polyps, along with a subepithelial protrusion in the distal rectum, which was approximately 0.7 cm in size, with erosive changes of the overlying mucosa ( Fig. 1 **a** ). The patient underwent endoscopic polypectomy 1 week later, at which time the erosive mucosa was noted to have recovered completely ( Fig. 1 **b** ). After 10 weeks, the patient underwent further tests, with white-light endoscopy now showing an ill-defined submucosal bulge with a convex polyp on its surface ( Fig. 2 **a** ). Endoscopic ultrasound (EUS) revealed a 5.2 × 3.1-mm heterogeneous mass originating from the submucosal layer ( Fig. 2 **b** ). The lesion was removed by ESD ( Video 1 ). Histologic analysis revealed acute and chronic inflammatory cells, foreign-body giant cells, plant-like matter, and convoluted hyaline rings, supporting the diagnosis of a pulse granuloma with a foreign-body reaction ( Fig. 3 ), consistent with a pulse granuloma. The patient was discharged following ESD, without any complications.

**Fig. 1:**
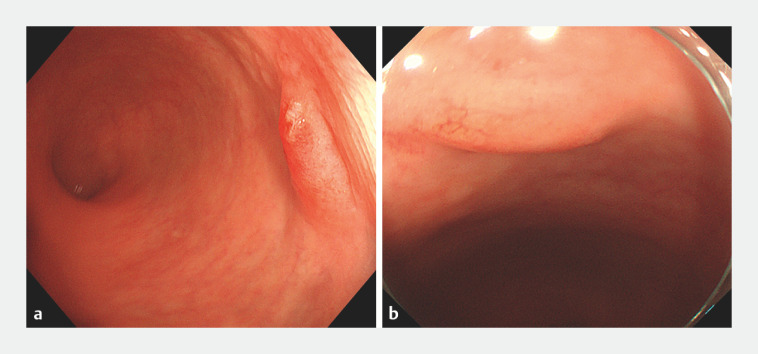
Endoscopic images showing a distal rectal subepithelial lesion: **a** as a subepithelial protrusion with erosive changes of the overlying mucosa; **b** 1 week later, with no evidence of the erosive overlying mucosa.

**Fig. 2:**
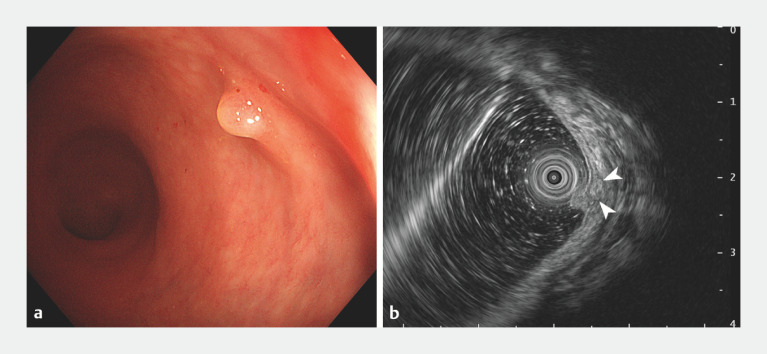
Appearance of the lesion 10 weeks later on: **a** colonoscopy, showing an ill-defined submucosal bulge with a polyp on its surface; **b** on endoscopic ultrasonography, showing a 5.2 × 3.1-mm heterogeneous mass originating from the submucosal layer (arrowheads).

**Fig. 3:**
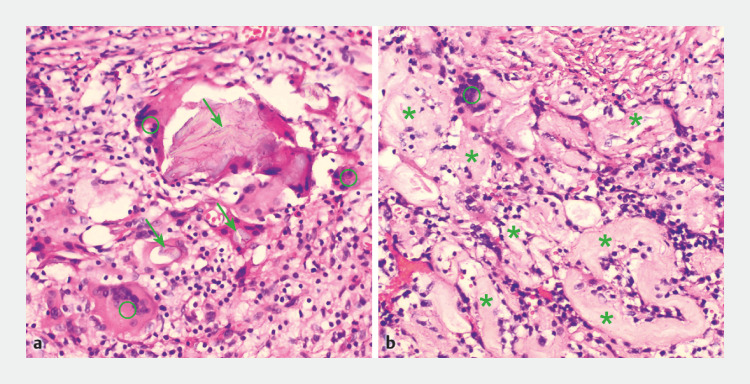
Histopathologic appearance of the resected lesion showing a granulomatous inflammatory process, with numerous foreign-body giant cells (circles), plant-like matter (arrows), and convoluted hyaline rings (stars), suggestive of a pulse granuloma (hematoxylin and eosin [H&E] staining, magnification × 200).

Endoscopic submucosal dissection of a subepithelial lesion initially seen as a subepithelial protrusion in the distal rectum on colonoscopy and confirmed to be originating from the submucosal layer on endoscopic ultrasound; histopathology of the resected specimen showed it to be a pulse granuloma.Video 1

Since it was first described in the lung in 1969 by Knoblich 2 , pulse granuloma has been reported in the oral and nasal cavity, skin, knee, fallopian tube and ovary, and intrahepatic portal vein 1 3 . It can also occur in the stomach, small intestine, colorectum, peritoneum, and mesentery 1 4 5 . A pulse granuloma is characterized by a chronic granulomatous reaction to a foreign body of vegetable origin 4 . In the present case, the mucosal damage seen above the lesion may have been the path by which the foreign bodies penetrated into the submucosal layer. As a rare lesion, familiarity with this entity’s distinctive histopathologic features may avoid a delayed diagnosis or misdiagnosis.

Endoscopy_UCTN_Code_CCL_1AD_2AG

## Notes

Endoscopy E-Videos https://eref.thieme.de/e-videos
*E-Videos* is an open access online section of the journal *Endoscopy* , reporting on interesting cases and new techniques in gastroenterological endoscopy. All papers include a high-quality video and are published with a Creative Commons CC-BY license. Endoscopy E-Videos qualify for HINARI discounts and waivers and eligibility is automatically checked during the submission process. We grant 100% waivers to articles whose corresponding authors are based in Group A countries and 50% waivers to those who are based in Group B countries as classified by Research4Life (see: https://www.research4life.org/access/eligibility/ ). This section has its own submission website at https://mc.manuscriptcentral.com/e-videos .
